# Characterization and Function of a Novel Welan Gum Lyase From Marine *Sphingomonas* sp. WG

**DOI:** 10.3389/fmicb.2021.638355

**Published:** 2021-02-09

**Authors:** Ai-Ping Chang, Jin Qian, Hui Li, Ying-Lu Wang, Jie-Ying Lin, Qiao-Mei He, Ya-Ling Shen, Hu Zhu

**Affiliations:** ^1^Engineering Research Center of Industrial Biocatalysis, Fujian Provincial Key Laboratory of Advanced Materials Oriented Chemical Engineering, Fujian Provincial Key Laboratory of Polymer Materials, Key Laboratory of OptoElectronic Science and Technology for Medicine of Ministry of Education, College of Chemistry and Materials Science, Fujian Normal University, Fuzhou, China; ^2^State Key Laboratory of Bioreactor Engineering, East China University of Science and Technology, Shanghai, China; ^3^Centre for Bioengineering and Biotechnology, China University of Petroleum (East China), Qingdao, China

**Keywords:** *Sphingomonas* sp. WG, welan gum, polysaccharide lyase, characterization, function

## Abstract

Welan gum, a kind of microbial exopolysaccharides, produced by the genus *Sphingomonas*, have great potential for application in many fields, such as the food industry, cement production, and enhanced oil recovery. But there are still challenges to reduce the cost, enhance the production and the quality. Herein, the bioinformatics analysis of *WelR* gene was preformed, and the characterization and function of WelR, welan gum lyase, from *Sphingomonas* sp. WG were investigated for the first time. The results indicated that 382nd (Asn), 383rd (Met), 494th (Asn), and 568th (Glu) were the key amino acid residues, and C-terminal amino acids were essential to keeping the stability of WelR. The optimal temperature and pH of the enzymatic activity were found to be 25°C and 7.4, respectively. And WelR was good low temperature resistance and alkali resistant. K^+^, Mg^2+^, Ca^2+^, Mn^2+^, and EDTA increased WelR activities, in contrast to Zn^2+^. Coupled with the change in glucose concentration and growth profile, the qRT-PCR results indicated that WelR may degrade welan gum existing in the culture to maintain bacterial metabolism when glucose was depleted. This work will lay a theoretical foundation to establish new strategies for the regulation of welan gum biosynthesis.

## Introduction

Sphingans, produced by the genus *Sphingomonas*, were important microbial exopolysaccharides (EPs), including gellan (S-60), welan (S-130), diutan (S-657), rhamsan (S-194), and so on. Due to high viscosity, biodegradability, and biocompatibility, these EPs have great potential for application in many fields, such as the food industry, cement production, and enhanced oil recovery (Li et al., 2016b). Among them, welan gum consisting of neutral sugars (mannose, glucose, and rhamnose in a molar ratio of 1:2.28:2.12), glucuronic acid, and some O-acyl groups exhibits excellent and attractive properties, such as pseudoplasticity, suspensibility, thickening property (Jansson et al., 1985; Xu et al., 2013; Li et al., 2016b). Welan gum could maintain its stability and high viscosity in aqueous solution at high temperature (20∼150°C), extreme acid or alkaline (pH = 2–12), and high salinity conditions (Plank, 2004; Li et al., 2013). Therefore, welan gum has potential industrial applications in food, concrete, ink, coating materials, and enhanced oil recovery (Kang et al., 1983; Sakata et al., 1996; Gao, 2016). Over the past decades, although many studies on screening of welan gum-producing strains and fermentation optimization have been carried out (Li et al., 2010, 2012; Zhang et al., 2016), there are still challenges to reduce the cost, enhance the production and the quality. Hence it will be of great significance to analyze the biosynthetic pathway of welan gum.

Polysaccharide lyases (EC 4.2.2.x) belong to a group of polysaccharide cleaving enzymes. They depolymerize certain acidic polysaccharides by a β-elimination mechanism on (1,4) glycosidic bonds (Michaud et al., 2003). Related studies show that there are some associations between the synthesis of polysaccharides and polysaccharide lyase. Presumably, polysaccharide lyase can control the molecular weight of polysaccharide and regulate medium viscosity for metabolite diffusions (Hatch and Schiller, 1998) or polysaccharide release (Boyd et al., 1993). So far, the β-eliminative cleavage enzyme has been widely described for application in food technology, therapeutic tools, polysaccharide structure analysis, protoplasts formation, oligosaccharide production, and especially control of polysaccharide rheological properties (Boyd et al., 1993; Hatch and Schiller, 1998; Michaud et al., 2003). Giavasis et al. (2008) reported that the gellan lyase not only plays an important role in controlling gellan molecular weight but also is related to the potential plant pathogenicity of *Sphingomonas* bacteria. In consideration of the associated polysaccharides structural regulation mechanism, the investigation on the function of welan gum lyase may provide new insights into the fermentation strategies of welan gum production.

In our previous work, a high welan gum-producing strain, *Sphingomonas* sp. WG, was screened out. Genome sequencing and annotation work were completed (Li et al., 2016a). The *wel* cluster for welan gum biosynthesis was predicted in the genome of *Sphingomonas* sp. WG. The *welR* gene was predicted to encode the welan gum lyase that controls the secretion and chain length of welan gum. Herein, the *welR* gene was firstly analyzed by a series of bioinformatics methods to obtain the physicochemical properties and structural information of the encoded protein WelR. And WelR was successfully expressed in *Escherichia coli* BL21(DE3) and purified by nickel ion affinity chromatography for deep study. Moreover, based on the multiple sequence alignments, the differential amino acids of WelR, the 19th (Leu), 352nd (Phe), 382nd (Asn), 383rd (Met), 494th (Asn), 558th (Gln), and 568th (Glu), were individually mutated to alanine, and six mutants (named L19A, F352A, N382A, M383A, N494A, Q558A, E568A) were successfully constructed. Ultraviolet (UV) and fluorescence (FL) spectroscopy were used to analyze the structural differences between WelR and its mutants to identify the key amino acid residues. According to the three-dimensional structure model analysis of WelR, the cavity at C-terminus may be a substrate-binding pocket. And the Sodium Dodecyl Sulfate-polyacrylamide Gel Electrophoresis (SDS-PAGE) analysis results indicated that there may be a small amount of partially degraded WelR in the resulting enzyme solution. After several attempts, it was found that the truncated enzyme obtained after the removal of 72 amino acids from the C-terminal segment was no longer degraded. So to further study the effects of the C-terminus on WelR structure, and C-terminal 72 amino acids truncated enzyme (named WelR-C_72_) was constructed by gene cloning. Circular dichroism (CD) spectroscopy was used to analyze the secondary structure, and dynamic light scattering (DLS) was used to analyze the size distribution in water. Next the enzyme activities *in vitro* and biochemical characterization of WelR were detected by applying 3, 5-dinitrosalicylic acid (DNS) method (Qin et al., 2018). To investigate the function of WelR *in vivo*, the growth of *Sphingomonas* sp. WG in the fermentation medium was determined, and the glucose content was also measured. Then quantitative real-time polymerase chain reaction (qRT-PCR) was applied to compare the relative expression level of the *welR* gene under the conditions of sufficient and lack of carbon source to analyze the role of WelR further. This work is a preliminary demonstration of the characterization and function of WelR from *Sphingomonas* sp. WG, which will provide a theoretical basis for establishing new strategies to regulate welan gum biosynthesis.

## Materials and Methods

### Strains, Plasmids, Culture Conditions, and Chemicals

*Sphingomonas* sp. WG was deposited in the China Center for Type Culture Collection (CCTCC) under the number M2013161. The extraction and purification of welan gum was performed as previously described (Li et al., 2016b). *E*. *coli* DH5α, *E*. *coli* BL21 (DE3), and the plasmid pET-28a^(+)^ were previously deposited in our lab. Both *E*. *coli* strains were grown at 37°C in LB broth or on LB broth agar (LB broth supplemented with 1.5% agar) supplemented with appropriate antibiotics when necessary.

DNA polymerase, restriction enzymes, T4 DNA ligase, standard molecular weight markers, RNAiso Plus, PrimeScript RT Reagent Kit with gDNA Eraser, and TB Green^TM^ Premix^®^ Ex Taq^TM^ II kit were purchased from TaKaRa Biotechnology (Dalian, China). Plasmid mini kit and gel extraction kit were obtained from Omega biotek (America). Primer synthesis and DNA sequencing were carried out by Shanghai Sangon Biotechnology (Shanghai, China). Other reagents were of analytical or higher grade and purchased from Sinopharm Chemical Reagent Co., Ltd. (Shanghai, China).

### Bioinformatics Analysis of WelR

The accession number of the whole genome sequence of *Sphingomonas* sp. WG is NZ_LNOS00000000 in the NCBI database. The accession number of the genome sequence of *WelR* is KTF67430.1. The nucleotide sequence of the *welR* was translated into the amino acid sequence using the software NovoPro^1^. The physical and chemical properties of the protein were analyzed by ProtParam^2^ (Wilkins et al., 1999), including predicted molecular weight (*M*_w_), number and composition of amino acids, theoretical *p*I (isoelectric point) and instability index. Hydrophilicity predictions were performed by ProtScale^3^ (Wilkins et al., 1999). Protein sequences similar to WelR were retrieved from the GenBank database using the BLAST algorithm on the National Center for Biotechnology Information (NCBI) server^4^. The amino acid sequence was aligned with homologous sequences using the MEGA6 program (Tamura et al., 2013) to predict key amino acids in the WelR. Secondary structures and domains of WelR were analyzed using the SOPMA server^5^ (Geourjon and Deleage, 1995) and the Simple Modular Architecture Research Tool (SMART) on the SMART server^6^ (Letunic et al., 2015), respectively. The WelR protein sequence was submitted to the SWISS-MODEL structure prediction website. The template proteins that were more than 30% consistent with the WelR sequence could not be retrieved from the protein database (PDB), indicating that the homology modeling method was not suitable for WelR structure modeling. So the tertiary structure was modeled by the online software Phyre2^7^ (Kelley et al., 2015) with PL6 family alginate lyase AlyGC (PDB: 5GKD, 19% identity) (Supplementary Figure 1A) as a template using fold recognition method. The PyMOL Molecular Graphics System was used to visualize the model.

### Cloning, Expression, and Purification of WelR

The *welR* gene was amplified by polymerase chain reaction (PCR) using genomic DNA of *Sphingomonas* sp. WG as the template and two synthetic oligonucleotides as primers. The oligonucleotides were 5′-GGAATTCATGCTTACCATGCCGGACG-3′ and 5′-CCCAAGCTTTCAGACGTGGTGCAATTCC-3′ with *Eco*R I and *Hin*d III sites in the 5′ region, respectively. The PCR reaction conditions were as follows: initial denaturation at 98°C for 8.0 min; 30 cycles of denaturation at 98°C for 10 s, annealing at 58°C for 5 s and extension at 72°C for 15 s; a final extension at 72°C for 10.0 min. PCR products of the expected size were purified and digested with *Eco*R I and *Hin*d III, and then ligated the vector pET-28a^(+)^ digested by the same two restriction endonucleases. Recombinant plasmids were transformed into *E*. *coli* DH5α to select transformants. The recombinant plasmid was sequenced to ensure sequence accuracy and designated as pET28a^(+)^-*welR*. To express the recombinant protein, the plasmid pET28a^(+)^-*welR* was transformed into *E*. *coli* BL21(DE3) competent cells by chemical transformation. The recombinant *E*. *coli* was grown to an OD_600_ of 0.6–0.8 in 100 mL LB medium containing 50 μg/mL kanamycin on a shaking incubator at 37°C and 180 rpm. The expression of WelR was induced using 0.4 mM isopropyl β-dthiogalactopyranoside (IPTG), and the cells were incubated for 20 h at 16°C. Next, the cells were harvested by centrifugation at 10,000 × g at 4°C for 10 min and washed twice with pre-chilled saline, resuspended in lysis buffer (50 mM NaH_2_PO_4_, 300 mM NaCl, 20 mM imidazole, pH 8.0). The pellets were disrupted using an ultrasonicator (SCIENTZ-IID, Ningbo Scientz Biotechnology Co. Ltd, China) to obtain total protein. Subsequently, the cell homogenate was centrifuged at 10,000 × g at 4°C for 10 min. The supernatant was loaded on the Ni-NTA-Sefinose column (Sangon Biotechnology, China) equilibrated with lysis buffer to purify the expressed (His)_6_-tagged soluble WelR. The column was washed with wash buffer (50 mM NaH_2_PO_4_, 300 mM NaCl, 50 mM imidazole, pH 8.0). Finally, the recombinant enzyme WelR was eluted with elution buffer (50 mM NaH_2_PO_4_, 300 mM NaCl, 100 mM imidazole, pH 8.0). The concentration of the purified enzyme eluted from the nickel column was ∼100–120 μg/mL. The yield of the WelR enzyme was about 40–48 mg/L. The eluted fractions were collected for secondary structure analysis. And the elution buffer of WelR was replaced by NaH_2_PO_4_-Na_2_HPO_4_ buffer solution (0.1 M, pH 8.0) using the ultrafiltration centrifuge tube for further enzyme activity assay. And the concentrated enzyme buffer solution was stored at −80°C.

### Alanine Scanning Mutation of WelR

According to the predicted results, a series of mutants were constructed using alanine-scanning site-directed mutagenesis by replacing the predicted key amino acids (L19, F352, N382, M383, N494, Q558, E568). All the primers used in this study were listed in Supplementary Table 1. The recombinant pET28a^(+)^-*welR* plasmid was used as the template. *E*. *coli* DH5α and *E*. *coli* BL21(DE3) strains were used for plasmid construction and protein expression, respectively. The expression and purification of protein mutants were performed as previously mentioned.

The structures of WelR and mutant proteins were analyzed by UV and FL spectroscopy. The concentrations of all proteins were similar but different, ranging from 80 to 110 μg/mL. Specifically, the concentrations of WelR, L19A, F352A, N382A, M383A, N494A and E568A were 110.5, 108.8, 96.4, 82.3, 83.4, 87.7, and 100.6 μg/mL, respectively. The solutions were an elution buffer. UV-visible absorption spectra were measured on a UV-visible spectrophotometer (TU-1810, Beijing Purkinje General Instrument Co. Ltd., China). FL spectra were recorded on an FS5 fluorescence spectrometer (Edinburgh Instruments, England), and the wavelength of the excitation light was 282 nm.

### Preparation of Truncated Enzyme WelR-C_72_

The primers 5′-GGAATTCATGCTTACCATGCCGGACG-3′ and 5′-AACAAGCTTTCACATCAGCAACAGCGCG-3′ were used to amplify welR-C_72_ gene from the genome of *Sphingomonas* sp. WG. The expression and purification of the truncated enzyme WelR-C_72_ were carried out in the same way as described in purifying WelR.

### Analysis of the WelR and WelR-C_72_

#### Sodium Dodecyl Sulfate-Polyacrylamide Gel Electrophoresis (SDS-PAGE)

Total protein and purified enzymes (including mutant proteins) were analyzed by SDS-PAGE using an 8% acrylamide slab gel. The protein was stained with Coomassie Brilliant Blue G-250. The molecular weight was determined using standard molecular weight markers as a reference.

#### Secondary Structure Analysis by CD Spectroscopy

CD spectra were measured on a Chirascan qCD spectrometer (Applied Photophysics Ltd., England) using a quartz cell with a 0.5 mm path length at room temperature in 180–260 nm. The concentrations of WelR and WelR-C_72_ were about 120 and 100 μg/mL, respectively. The solutions were 20 mM Tris-HCl buffer (pH 8.0). The CD spectra were obtained after averaging of three scans and subtraction of the background. The spectra were analyzed by K2D3 online tools^8^ (Louis-Jeune et al., 2012). Results were expressed as mean residue ellipticity (MRE) [(θ) deg⋅cm^2^⋅dmol^–1^].

#### Size Distribution Analysis by DLS

DLS was measured using the Zetasizer Nano ZSE particle size analyzer (Malvern Instruments, England) at 25°C. The concentration of WelR and WelR-C_72_ was about 100 μg/mL. The solutions were an elution buffer.

### Enzyme Activity Assay

The protein concentration was estimated using the Lowry method (Lowry et al., 1951). The reaction was initiated by adding 400 μL of WelR enzyme solution (∼1.5 mg/mL) or water (blank control) to 1 mL 0.5% (w/v) welan gum aqueous solution. The enzymatic reaction was carried out for several hours at 40°C. The viscosity changes of the two solutions were recorded by photographs. And the reducing sugars were measured using the DNS method with D-glucose as standard (Supplementary Figure 2; Miller, 1959). The specific experimental steps of the DNS method were as follows: Briefly, 100 mL DNS solution was prepared by mixing 18.2 g potassium sodium tartrate, 0.63 g dinitrosalicylic acid, 2.1 g sodium hydroxide, and 0.5 g phenol. The solution was used after being stored for a week in the dark. 200 μL reaction solution and 300 μL DNS solution were mixed and then heated at 100°C for 5 min to develop the red-orange color. Upon cooling to ambient temperature, the UV-vis absorbance of the stabilized solution was measured on a Synergy-H1 microplate reader (Bio-Tek, United States) at 540 nm. The reaction solution without enzyme was used as a blank control.

### Effect of Temperature, pH, Metal Ions, and Chelators on Enzymatic Activity of WelR

The optimal temperature of the enzymatic activity was determined by measuring the activity at various temperatures from 15 to 45°C. The thermostability of WelR was evaluated by measuring the residual activity after incubation at 4–45°C for 1 h. The optimal pH of the enzymatic activity was determined by evaluating different buffers in the assay system at 30°C, including 100 mmol/L citrate buffer (pH 4.0–5.0), phosphate buffer (pH 5.8–6.4), Tris-HCl buffer (pH 7.4–9.0), and Na_2_HPO_4_-NaOH buffer (pH 10.0–11.0). To determine pH stability, the residual activity was measured after the enzyme was incubated in the pH range of 4–11 for 1 h at 30°C. To examine the influence of different metal ions and chelators, the residual activity of WelR was measured after incubation with various metal ions (KCl, MgSO_4_, ZnSO_4_, CaCl_2_, and MnCl_2_) and ethylene diamine tetraacetic acid (EDTA) at 1 mM for 1 h at 30°C. One unit (1 U) was defined as the amount of enzyme required to liberate 1 μmol of reducing sugar per minute. Repeat the above tests for three times.

In addition to pH effects, other enzymatic properties were tested using NaH_2_PO_4_-Na_2_HPO_4_ solution (pH 8.0). In addition to temperature effects, other enzymatic reactions were carried out at 30°C. The reaction was initiated by adding 100 μL of WelR enzyme solution to 400 μL 0.5% (w/v) welan gum solution, and the enzymatic reaction was carried out for 3 h. Approximately 500 μg/mL enzyme solution was used for the optimal temperature, and thermostability assay, ∼300 μg/mL enzyme solution was used for the optimal pH assay, ∼400 μg/mL enzyme solution was used for the pH stability assay, ∼350 μg/mL enzyme solution was used for the influence of different metal ions and chelators assay.

### Expression Level Assay by qRT-PCR

*Sphingomonas* sp. WG was inoculated to the fermentation medium at a ratio of 10% (v/v) and cultured for 75 h after cultivating the seeds. Next, the total RNAs of *Sphingomonas* sp. WG grown under different conditions was extracted using RNAiso Plus and was used as a template to synthesize cDNA using the PrimeScript^®^ RT Reagent Kit with gDNA Eraser. Following cDNA synthesis, qRT-PCR was performed using a TB Green^TM^ Premix Ex Taq^TM^ II kit and monitored with a CFX96-Touch instrument (Bio-Rad, United States). The 16S rRNA gene was selected as the reference gene (Li et al., 2018). All the primers are listed in Supplementary Table 1. Each 25-μL PCR system contained 12.5 μL of TB Green^TM^ Premix Ex Taq^TM^ II, 1.0 μL of the forward and reverse primers (10 μmol L^–1^), 2.0 μL of the cDNA template and 8.5 μL of sterilized water. The qRT-PCR was performed with an initial denaturation at 95°C for 30 s followed by 40 amplification cycles of 95°C for 5 s and 60°C for 30 s. After the amplification, a melting curve was generated with a temperature gradient of 0.1°C/s from 60 to 95°C to confirm the specificity of each product. The expression of the *welR* gene under the sufficient carbon source condition was used as the reference, and the relative changes in gene expression were calculated using the 2^–ΔΔCq^ method (Livak and Schmittgen, 2001). Each sample was assessed in three replicates.

During fermentation, samples were withdrawn periodically and diluted appropriately. The content of glucose in the culture broth was assayed by DNS method (Miller, 1959). The optical density was measured at 600 nm to assess the growth rate after diluting the fermentation broth appropriately and the growth curve of *Sphingomonas* sp. WG was subsequently plotted.

## Results and Discussion

### Bioinformatics Analysis of the Welan Gum Lyase WelR

According to ProtParam and ProtScale, the *welR* was 2,028 bp in length and encoded a protein (WelR) consisting of 675 amino acid residues (Supplementary Figure 3). Its amino acid composition and physic-chemical parameters are shown in Supplementary Table 2 and Table 1. The highest and lowest amino acid in WelR were serine (S, 72, 10.7%) and tryptophan (W, 6, 0.9%), respectively. There was no disulfide bond in the structure of WelR because of no cysteine. There were 52 negatively charged residues (Asp, Glu) and 48 positively charged residues (Arg, Lys) in WelR. Its grand average of hydropathicity was −0.008, which indicated that WelR was a hydrophilic protein. The molecular weight and theoretical *p*I of WelR were 71 kDa and 6.17. The instability index (II) was computed to be 29.39, indicating that WelR was a stable protein.

**TABLE 1:** Physic-chemical property analysis of WelR.

Characterizations	Parameter
Number of amino acids	675
Molecular weight	71044.94
Theoretical *p*I	6.17
Negatively charged residues (Asp + Glu)	52
Positively charged residues (Arg + Lys)	48
Instability index (II)	29.39

Subsequently, Multiple sequence alignments were performed using the MEGA6 software to identify key amino acids in WelR (Supplementary Figure 4). The results indicated that the amino acids unique to the 19th (Leu), 352nd (Phe), 382nd (Asn), 383rd (Met), 494th (Asn), 558th (Gln), and 568th (Glu) may be the key amino acid residues of WelR. SOPMA and SMART were applied to predict the secondary structures. The percentages of alpha-helix, extended strand, beta-turn, and random coil in WelR were about 16.89, 34.96, 9.04, and 39.11, respectively (Supplementary Figure 5). Structural domain analysis revealed that WelR contains no signal peptide, but four parallel beta-helix repeated (PbH1) domains, a Laminin_G_3 superfamily, and two low complexity regions (LCR) (Supplementary Figure 6). The PbH1 domains of WelR covered sites 99–264, with the function of polysaccharide hydrolysis (Jenkins et al., 1998). Its Laminin_G_3 superfamily spanned sites 421–570, which belonged to the Concanavalin A-like lectin/glucanases superfamily. This superfamily includes a diverse range of carbohydrate-binding domains and glycosyl hydrolase enzymes that share a common structure (Lu et al., 2020). These functional domains indicated that WelR have the function of degrading welan gum. Phyre2 results showed that the WelR has an open structure with a central cavity, and the inner diameter of the cavity at C-terminus was larger, which may be a substrate-binding pocket (Supplementary Figure 1B).

### Expression and Purification of Recombinant Proteins

**FIGURE 1:**
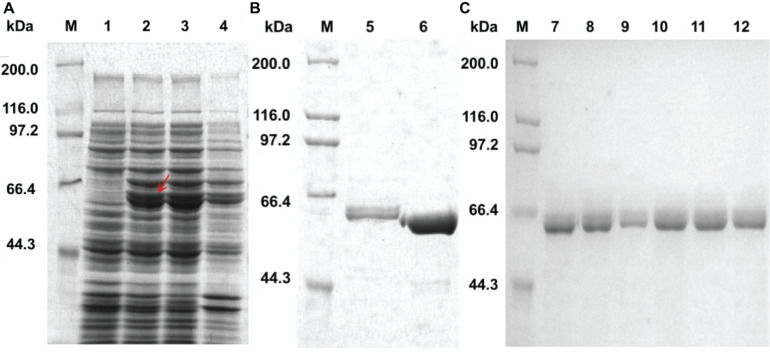
Expression and purification of WelR, WelR-C_72_ and mutants. **(A)** Expression analysis of recombinant WelR. **(B)** Purified WelR and C-terminal truncation enzyme WelR-C_72_. **(C)** Purification of WelR mutants using a Ni-NTA sefinose column. M: Molecular weight marker; Lane 1: total protein of *E. coli* BL21/pET-28a^(+)^; Lane 2–4: total protein, soluble protein, and insoluble protein of *E. coli* BL21/pET28a^(+)^-welR induced by 0.4 mM IPTG at 16°C for 20 h; Lane 5–12: purified WelR, WelR-C_72_, L19A, F352A, N382A, M383A, N494A, and E568A.

The *welR, welR-C_72,_* and mutant genes were expressed in the *E*. *coli* BL21(DE3)-pET28a^(+)^ system. Most of the recombinant WelR existed as soluble body forms after induction with 0.4 mM IPTG at 16°C for 20 h (Figure 1A). The purified WelR was detected by SDS-PAGE, and there were two similar size bands near the 60 kDa after nickel ion affinity chromatography (Figure 1B). The smaller one was presumably the partially degraded WelR, for these two were always eluted at the same imidazole concentration. A C-terminally truncated enzyme WelR-C_72_ was obtained after truncating C-terminal 72 amino acids, there was one band in Figure 1B, indicating the truncated enzyme obtained after the removal of 72 amino acids from the C-terminal segment was no longer degraded. By using Ni-NTA sefinose affinity chromatography, the mutants were purified (Figure 1C) except Q558A. As shown in Supplementary Figure 7, after elution with different concentrations of imidazole elution buffer, the expressed Q558A were not obtained. So we guessed the reason may be that its (His)_6_-tag was folded inside and Q558A was unable to bind to the nickel column. The molecular weight of the mutants was estimated to be about 60 kDa by SDS-PAGE, which was in good agreement with their theoretical value.

### Determination of Key Amino Acid Residues of WelR

**FIGURE 2:**
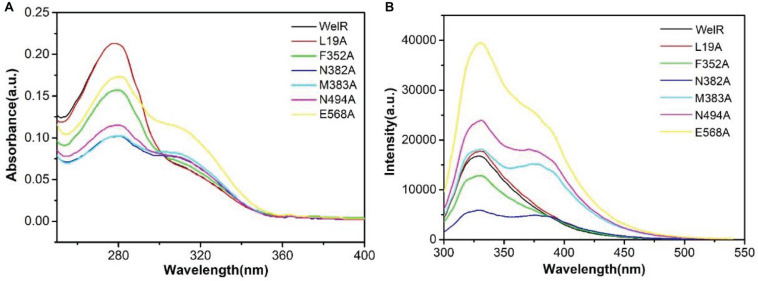
UV absorption spectra **(A)** and FL spectra **(B)** of the WelR and mutants. The concentrations of WelR, L19A, F352A, N382A, M383A, N494A, and E568A were 110.5, 108.8, 96.4, 82.3, 83.4, 87.7, and 100.6 μg/mL, respectively.

In Figure 2A, there was only a strong UV absorption peak at 280 nm in WelR and mutants L19A, F352A, which was mainly caused by π→π^∗^ and n→π^∗^ transitions of tryptophan and tyrosine aromatic heterocycles (Cheng and Zhang, 2008), indicating that the structure of L19A and F352A was similar to WelR. The other four mutants (N382A, M383A, N494A, and E568A) had a gentle side peak near 310 nm in addition to the peak at 280 nm. The change in peak position was due to a change in the microenvironment of aromatic amino acids, which indicated that the conformation of the N382A, M383A, N494A, and E568A had changed significantly, comparing to the wild WelR.

From FL spectra (Figure 2B), the maximum emission peak of WelR and two mutants (L19A and F352A) was shown at 328 nm, corresponding mainly to tryptophan residues in the hydrophobic region, suggesting that the structure of L19A and F352A was similar to WelR. In addition to the peak at 328 nm, there was a weak fluorescence emission peak at 375 nm in mutants N382A, M383A, N494A, and E568A. The emission peak will red-shift as the polarity increases when the tryptophan residue moves from the inside of the protein to the hydrophilic surface (Burshtein et al., 1973). So the fluorescence peak at 375 nm corresponds to the tryptophan in a hydrophilic environment. These results were consistent with the results of UV spectra. The conformations of L19A and F352A had hardly changed, for leucine and phenylalanine are both non-polar amino acids like alanine, which may have little effect on the folding of the protein. However, Asn382, Met383, Asn494, and Glu568 are polar or weakly polar amino acids. We concluded that these amino acids, which are mutated to alanine, would tend to fold inside the hydrophobic interior of the protein, causing a part of tryptophan to be exposed to the polar surface. So it appears that 382nd (Asn), 383rd (Met), 494th (Asn), and 568th (Glu) are the key amino acid residues of WelR, which play a critical role in maintaining the correct conformation of WelR.

### Effects of C-Terminal Residues on WelR

**FIGURE 3:**
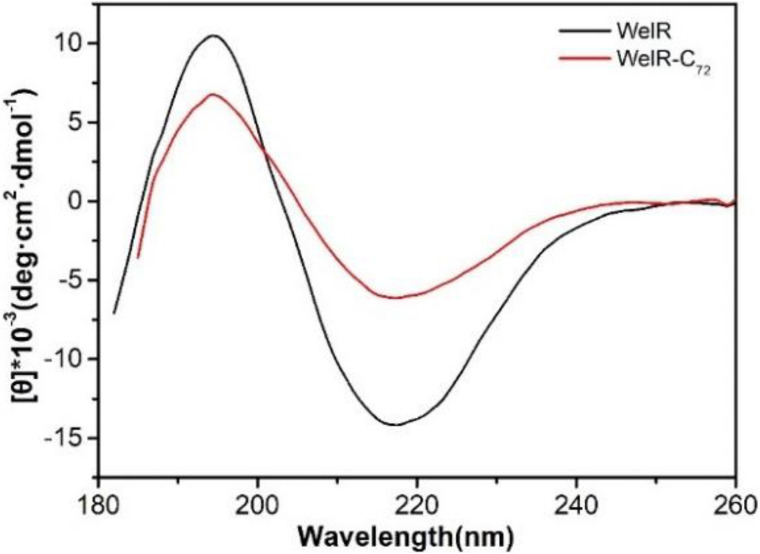
CD spectra of WelR and WelR-C_72_. The concentrations of WelR and WelR-C_72_ were about 120 and 100 μg/mL, respectively.

Far-UV CD spectroscopy from 180 to 260 nm was carried out to measure the secondary structures of WelR and WelR-C_72_. The results showed that WelR and WelR-C_72_ have a similar CD spectrum with a positive maximum around 195 nm and a negative minimal around 217 nm (Figure 3), which is a typical feature of beta-sheet rich protein. The secondary structure was calculated by uploading data to the website http://cbdm-01.zdv.uni-mainz.de/~andrade/k2d3/. The alpha-helix, beta-sheet, and random coil contents of the WelR were about 18.93, 25.87, and 55.2%, while those of the WelR-C_72_ were about 1.98, 32.86, and 65.16%, respectively. These results suggested that the content of alpha-helix obviously decreases; the proportion of beta-sheet and random coil increases after truncating 72 amino acids at the C-terminus of WelR. From this, the presence of C-terminal amino acids of WelR is indispensable for maintaining its ordered structure.

**FIGURE 4:**
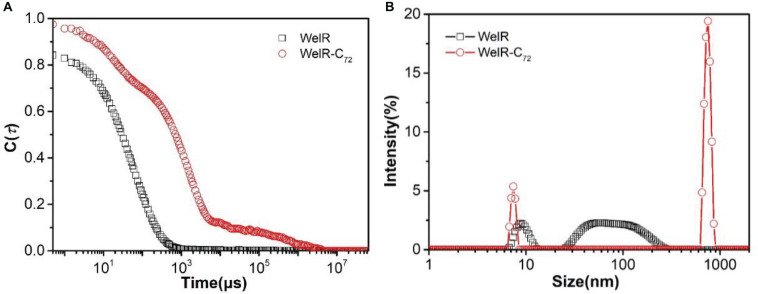
DLS intensity autocorrelation function **(A)** and size distribution by intensity **(B)** of WelR and WelR-C_72_. The concentration of WelR and WelR-C_72_ was about 100 μg/mL.

The DLS technique was then applied to study the size distribution of WelR and WelR-C_72_ in aqueous solution. In Figure 4A, the curve of the intensity autocorrelation function of WelR was smooth and anti-S shaped with once decay. In contrast, the curve of WelR-C_72_ was rough with increased decay time and decay times, suggesting that there were polydisperse aggregates in the WelR-C_72_ solution (Soraruf et al., 2014). The size distribution by intensity (Figure 4B) also confirmed this argument: 90.5 nm 81%, 9.2 nm 19% for WelR, while 746.9 nm 82%, 7.4 nm 18% for WelR-C_72_. WelR-C_72_ was more likely to aggregate in water, indicating the removal of the C-terminal amino acids changes the protein-protein interaction and disrupts the stability of the colloidal solution.

### Activity Analysis of WelR *in vitro*

**FIGURE 5:**
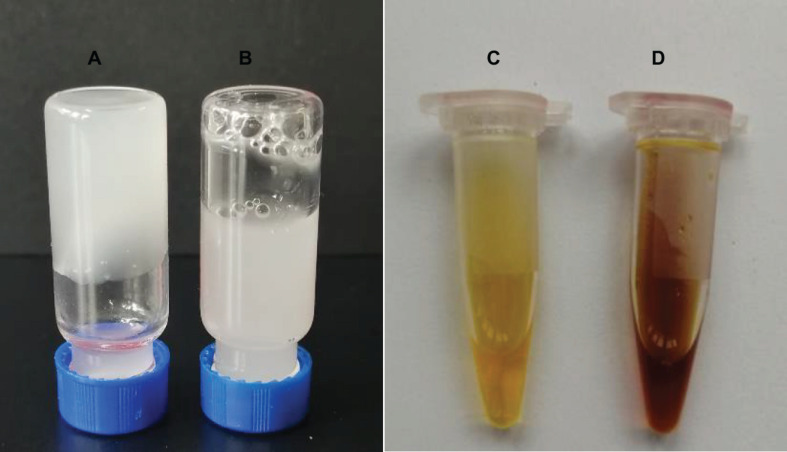
Activity analysis of WelR *in vitro*. Photographs of 0.5% (w/v) welan gum solution after 10 h of reaction without WelR **(A)** and treated with DNS developing reagent **(C)** (blank control); with WelR **(B)** and treated with DNS developing reagent **(D)**.

A solution of 0.5% (w/v) welan gum formed a gel (the sample does not flow if turned upside down, as evident from Figure 5A). During the enzymatic reaction, WelR would attack the welan gum chains, resulting in the decrease of molecular weight and solution viscosity. As shown in Figure 5B, after 10 h of reaction in the presence of WelR, the initially rigid opaque gel turned to a fluid suspension, and there was no significant change in the blank control (Figure 5A), indicating that WelR, as a welan gum lyase, can degrade welan gum effectively. And the reducing sugars were measured by using DNS as color developing reagent. For the blank control (Figure 5C), the developing color was the original yellow. While the developing color of the solution after WelR-catalyzed reaction was brownish red (Figure 5D), indicating the reducing sugars were produced (Miller, 1959). Compared with the standard curve (Supplementary Figure 2), the yield of the reducing sugars was about 8%.

### Biochemical Characterization of WelR

**FIGURE 6:**
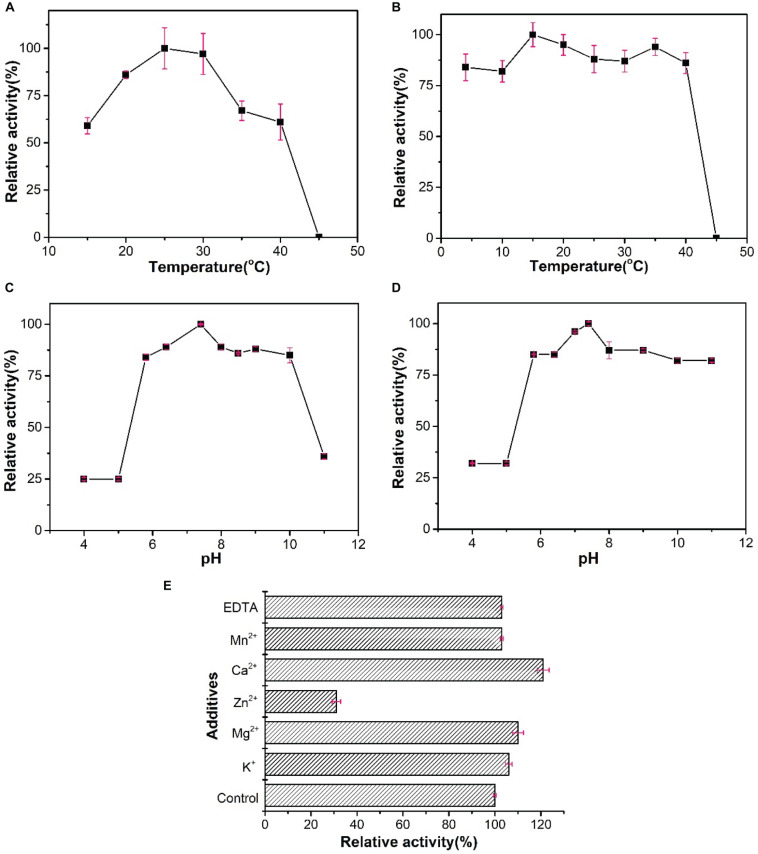
Biochemical characterization of WelR. **(A)** Activity of WelR over a range of temperatures (15–45°C). Activity at 25°C was taken as 100%. **(B)** Thermal stability of WelR. Activity after being incubated at 15°C was taken as 100%. **(C)** Activity of WelR over a range of pH values (4–11). Activity in pH 7.4 buffer was taken as 100%. **(D)** The pH stability of WelR. Activity after being incubated in pH 7.4 buffer was taken as 100%. **(E)** Effects of various metal ions and EDTA on the activity of WelR. Activity in the blank control was taken as 100%. Values are presented as the mean ± SD; *n* = 3.

The optimal temperature of WelR was 25°C, and it showed 59 and 85% of the maximal activity at 15°C and 20°C (Figure 6A), respectively, indicating that WelR was a cold-adapted welan gum lyase. At the same time, alginate lyase from *Vibrio* (Wang et al., 2013) and oligoalginate lyase from *Shewanella* (Li et al., 2016c) showed less than 40% of the maximal activity at 20°C. More than 75% of WelR activity remained after being incubated at 4–40°C for 1 h, but no enzyme activity remained after being incubated at 45°C (Figure 6B). These results indicated that WelR was a low temperature enzyme. When assayed in various pH values, the maximum activity of WelR was observed at pH 7.4 (Figure 6C). And WelR retained over 80% of initial activity after incubated at pH 5.8–11.0 for 1 h, 30% at pH 4.0–5.0 (Figure 6D), indicating WelR was alkali resistant but not acid resistant. This likely represents an evolutionary adaptation of the marine bacterium to the low temperature and weakly alkaline environment of seawater. This property is similar to that of ulvan lyase derived from marine bacterium *Alteromonas* sp. (Gao et al., 2019; Qin et al., 2020). Effects of metal ions (K^+^, Mg^2+^, Zn^2+^, Ca^2+^, Mn^2+^) and chelating agent (EDTA) are demonstrated in Figure 6E. All of the tested monovalent and divalent metal ions and EDTA increased WelR activities, in contrast to Zn^2+^. Ca^2+^ obviously enhanced enzymatic activity by 20%, and Zn^2+^ strongly inhibited enzymatic activity by 70%. Similarly, the activities of ulvan lyase from *Pseudoalteromonas* sp. strain PLSV (Qin et al., 2018) and alginate lyase from marine *Microbulbifer* sp. ALW1 (Zhu et al., 2016) was strongly inhibited in the presence of Zn^2+^. But the reason why Zn^2+^ strongly inhibited the activity of these lyases remains to be elucidated.

### Function Study of WelR *in vivo*

**FIGURE 7:**
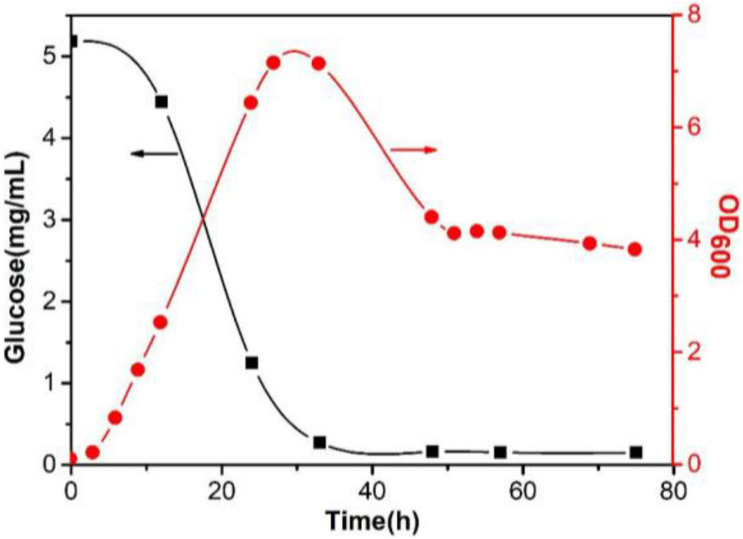
Glucose content change in the fermentation medium and the growth curve of *Sphingomonas* sp. WG.

In Figure 7, the concentration of glucose gradually decreased as fermentation proceeded, and the glucose in the medium was almost exhausted until 33 h. The growth curve of *Sphingomonas* sp. WG showed that the bacteria entered the logarithmic phase after a short period of lag phase, and reached the maximum density at 27 h. After a stationary phase of 6 h, the density of the cells decreased due to the depletion of the carbon source in the medium. After 48 h, the OD_600_ remained at a high level (∼4) with little change, meaning that the bacterial concentration could be maintained at a certain level even if there was no glucose in the fermentation medium. It was assumed that the metabolic pathways of *Sphingomonas* sp. WG may change when glucose was depleted.

**FIGURE 8:**
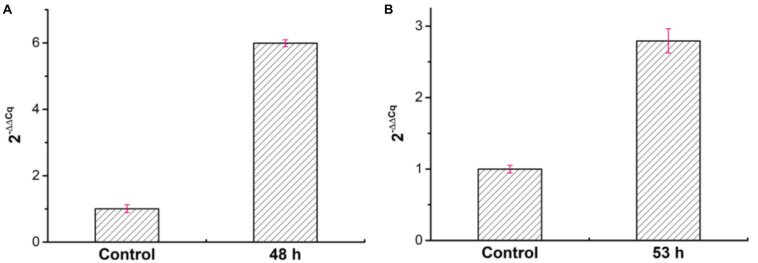
Relative expression levels of *welR* at 48 h **(A)** and 53 h **(B)** by using the 2^− ΔΔCq^ calculation method.

Besides, to analyze the expression of the *welR* gene, we took the cells cultured for 12 h under the condition of sufficient glucose as the control group, and the cells cultured for 48 and 53 h were used as the experimental groups under the condition of lack of glucose. As shown in Supplementary Table 3 and Figure 8, the qRT-PCR results showed that the expression levels of *welR* at 48 and 53 h were 5.99 and 2.79 times higher than that of 12 h, respectively, indicating that the expression of *welR* increased after glucose was depleted. These results also proved that WelR may degraded welan gum existing in the culture to maintain bacterial metabolism when glucose was insufficient.

## Conclusion

This work was the first attempt to study the characterization and function of welan gum lyase WelR of *Sphingomonas* sp. WG. It will be beneficial to establish new strategies for fermentation regulation and industrial production of welan gum. The specific WelR-catalyzed degradation mechanism and enzymatic products still need to be confirmed by a large number of experiments, which are worthy of further study.

## Data Availability Statement

All datasets generated for this study are included in the article/Supplementary Material, further inquiries can be directed to the corresponding author/s.

## Author Contributions

Y-LS and HZ: conceptualization. JQ and A-PC: investigation and writing—original draft preparation. HL and Y-LW: data curation. A-PC and HZ: writing—review and editing. All authors have read and agreed to the published version of the manuscript.

## Conflict of Interest

The authors declare that the research was conducted in the absence of any commercial or financial relationships that could be construed as a potential conflict of interest.

## Footnotes

1 http://www.novopro.cn/tools/translate.html

2 https://web.expasy.org/protparam/

3 https://web.expasy.org/protscale/

4 http://www.ncbi.nlm.nih.gov

5 https://npsa-prabi.ibcp.fr/cgi-bin/npsa_automat.pl?page=/NPSA/npsa_sopma.html

6 http://smart.embl-heidelberg.de/

7 http://www.sbg.bio.ic.ac.uk/phyre2/html/page.cgi?id=index

8 http://cbdm-01.zdv.uni-mainz.de/~andrade/k2d3/
