# Supplementary material for: Characterization and Function of a Novel Welan Gum Lyase From Marine Sphingomonas sp. WG
Source: Front Microbiol. 2021 Feb 9;12:638355. doi: 10.3389/fmicb.2021.638355 (PMC7899989; doi:10.3389/fmicb.2021.638355)
Supplement: Supplementary file 1 [file Data_Sheet_1.docx]

Supplementary Materials

## Supplementary Figures

**Supplementary Figure 1.** The three-dimensional structure of the template AlyGC (A) and WelR (B). The WelR protein sequence was submitted to the SWISS-MODEL structure prediction website. The template proteins that were more than 30% consistent with the WelR sequence could not be retrieved from the protein database (PDB), indicating that the homology modeling method was not suitable for WelR structure modeling. So the tertiary structure of WelR was modeled by the online software Phyre2 (http://www.sbg.bio.ic.ac.uk/phyre2/html/page.cgi?id=index) with PL6 family alginate lyase AlyGC (PDB: 5GKD, 19% identity) as a template using fold recognition method.


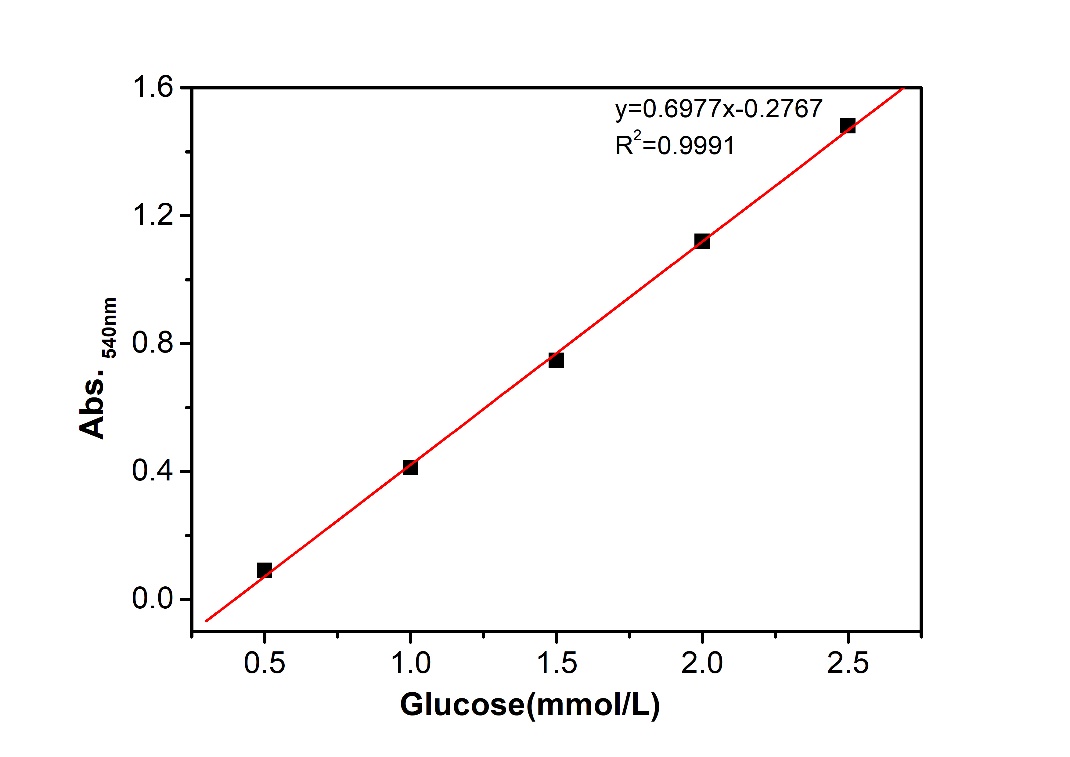


**Supplementary Figure 2.** Standard curve of DNS reagents.

**
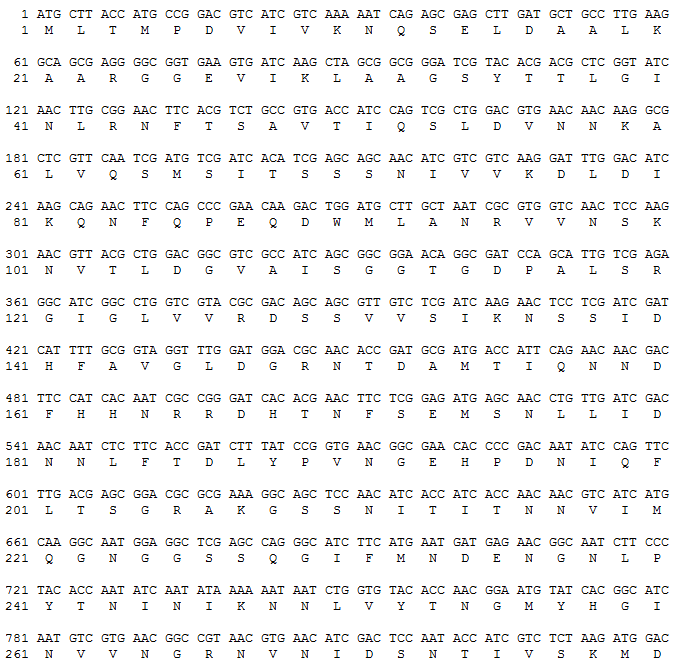

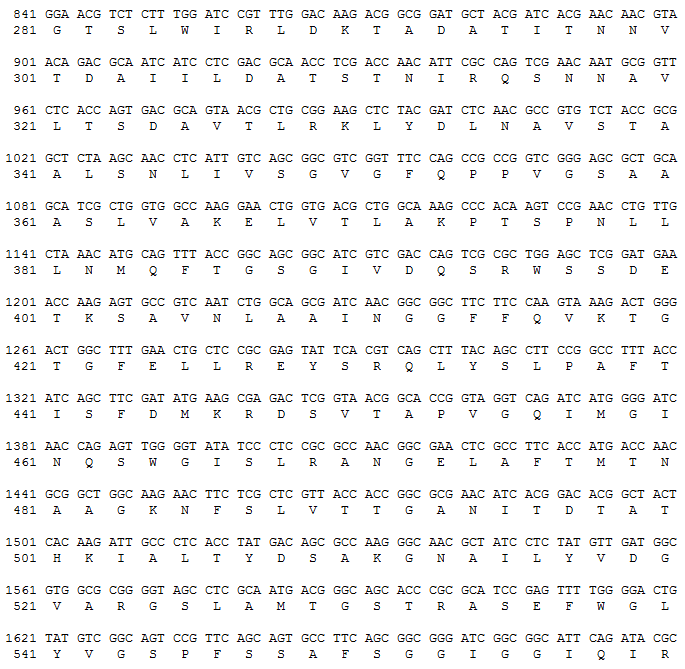

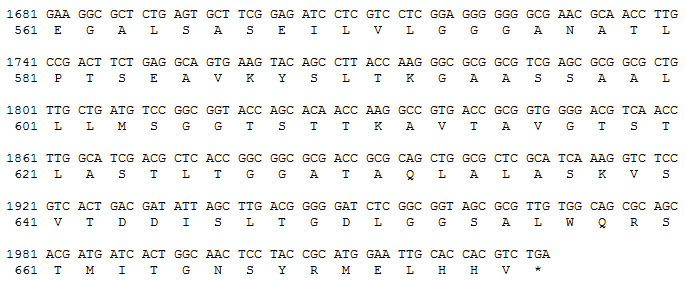
**

**Supplementary Figure 3.** The deduced amino acid sequence of WelR.

**
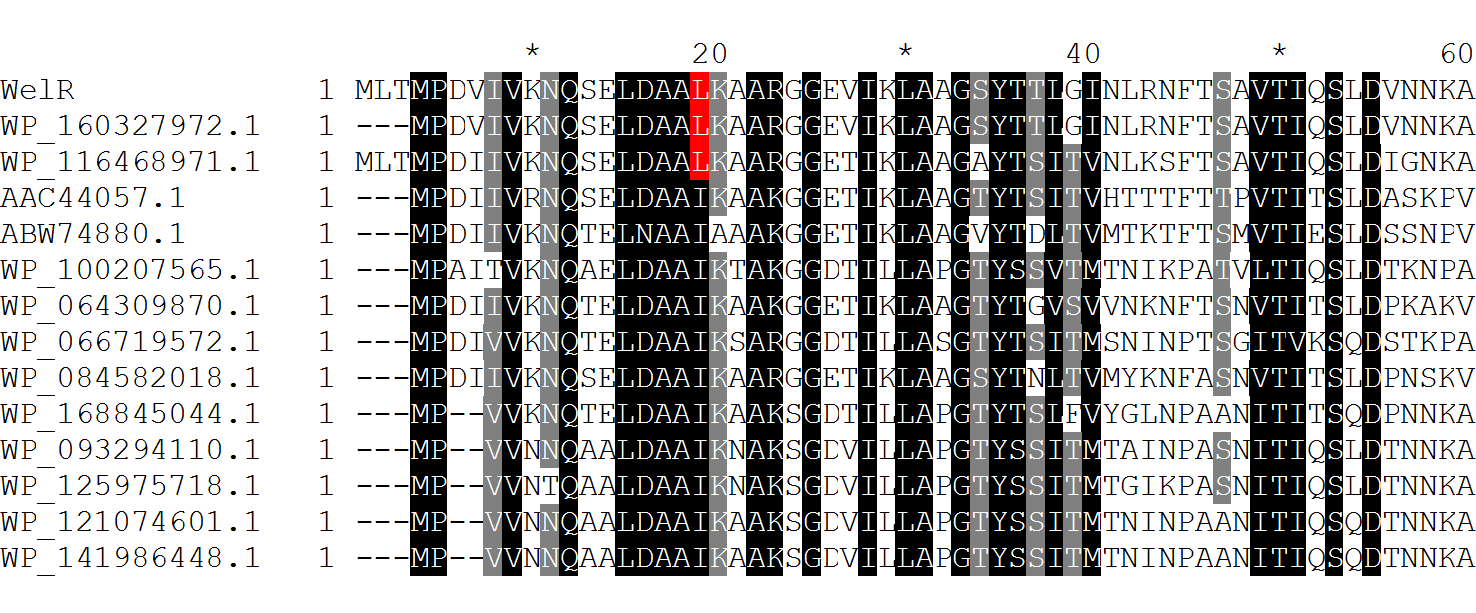

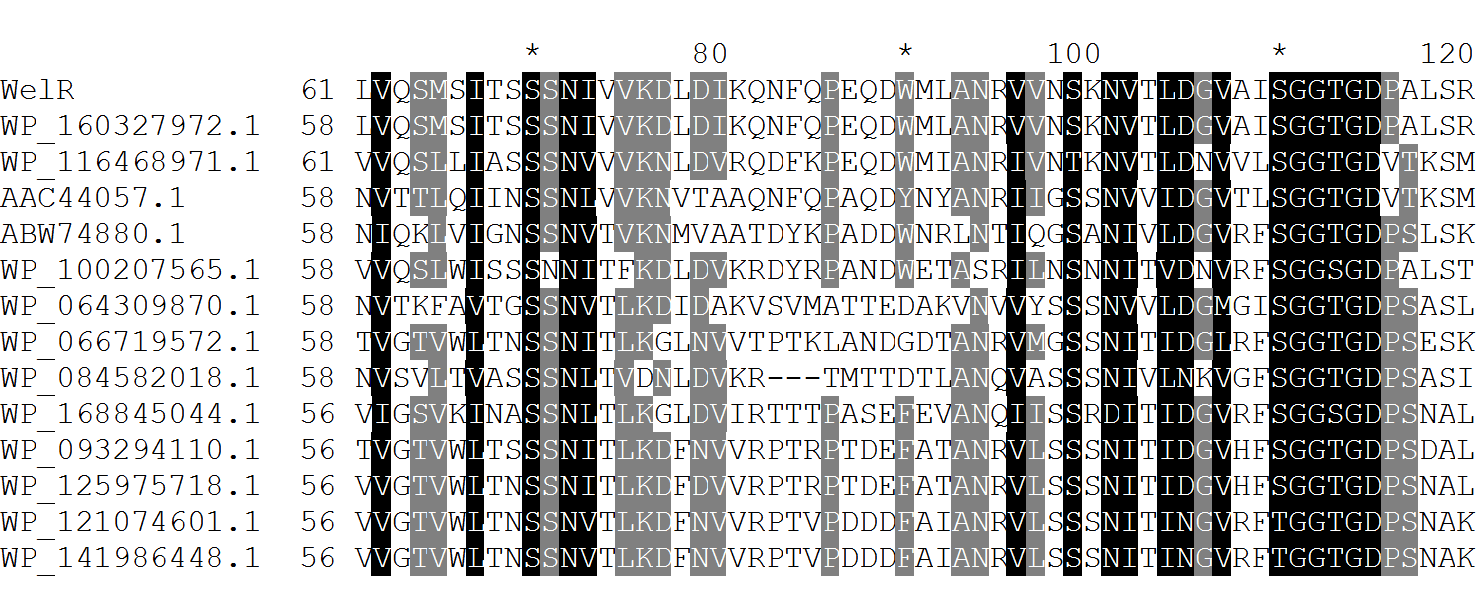

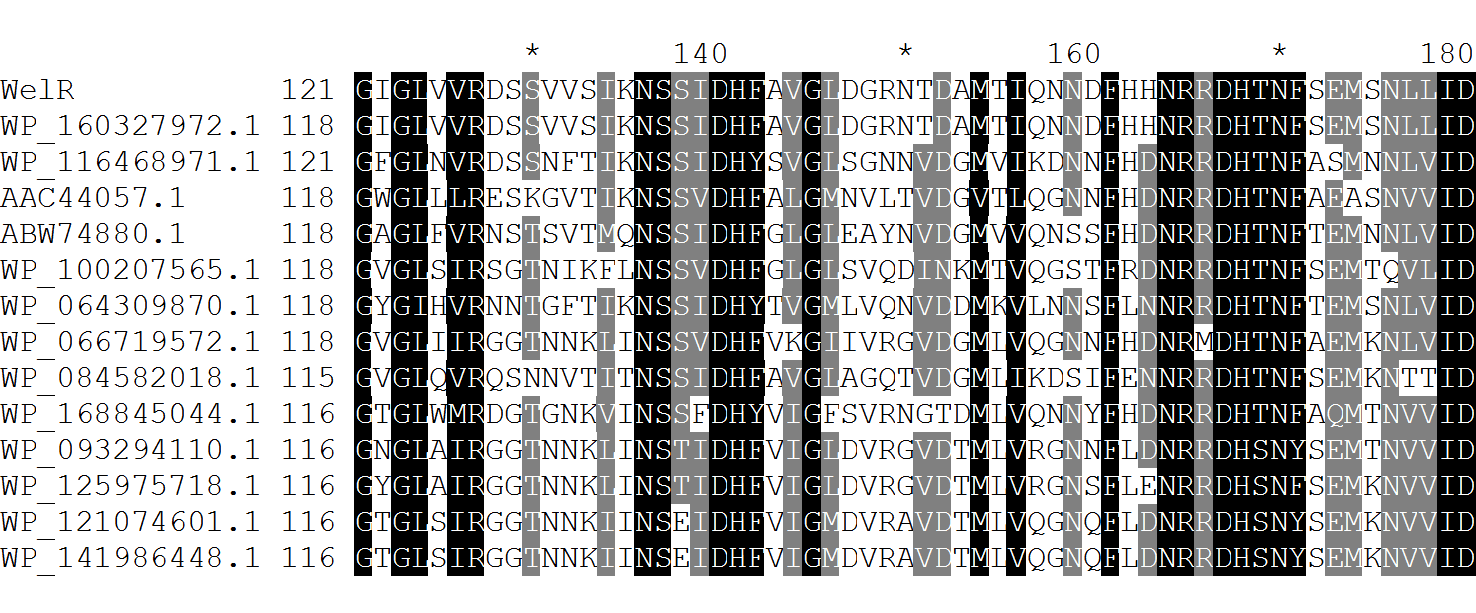

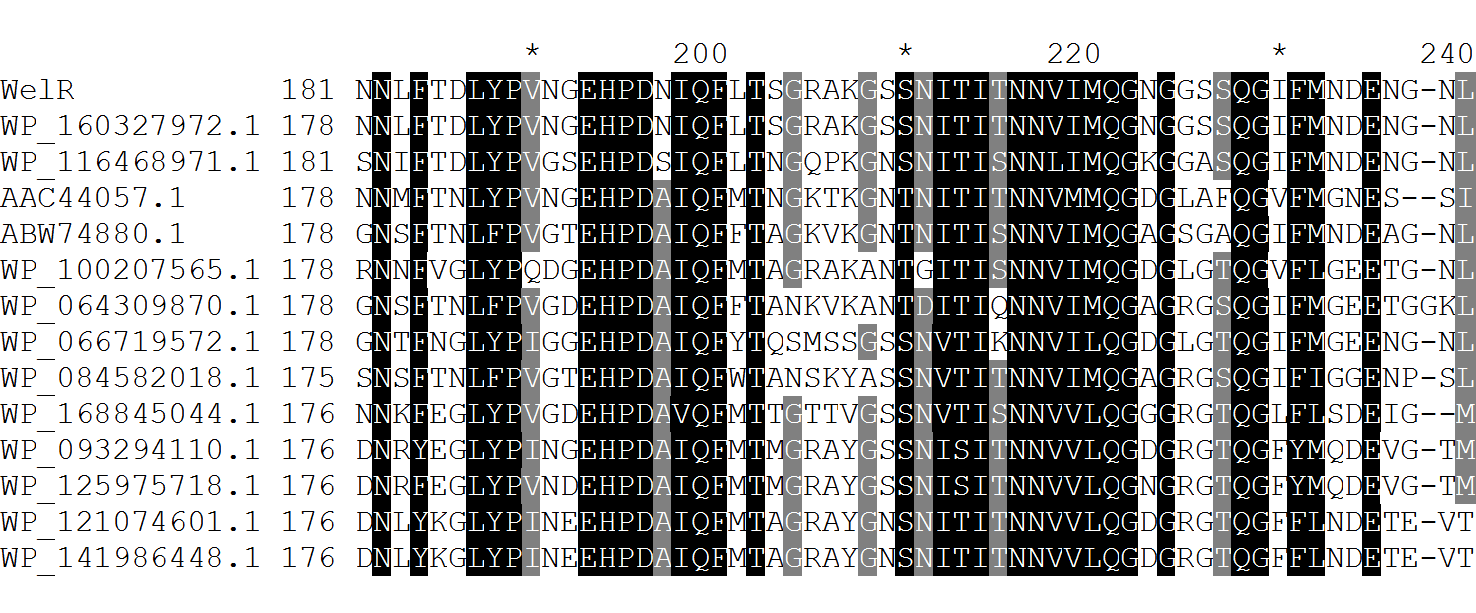

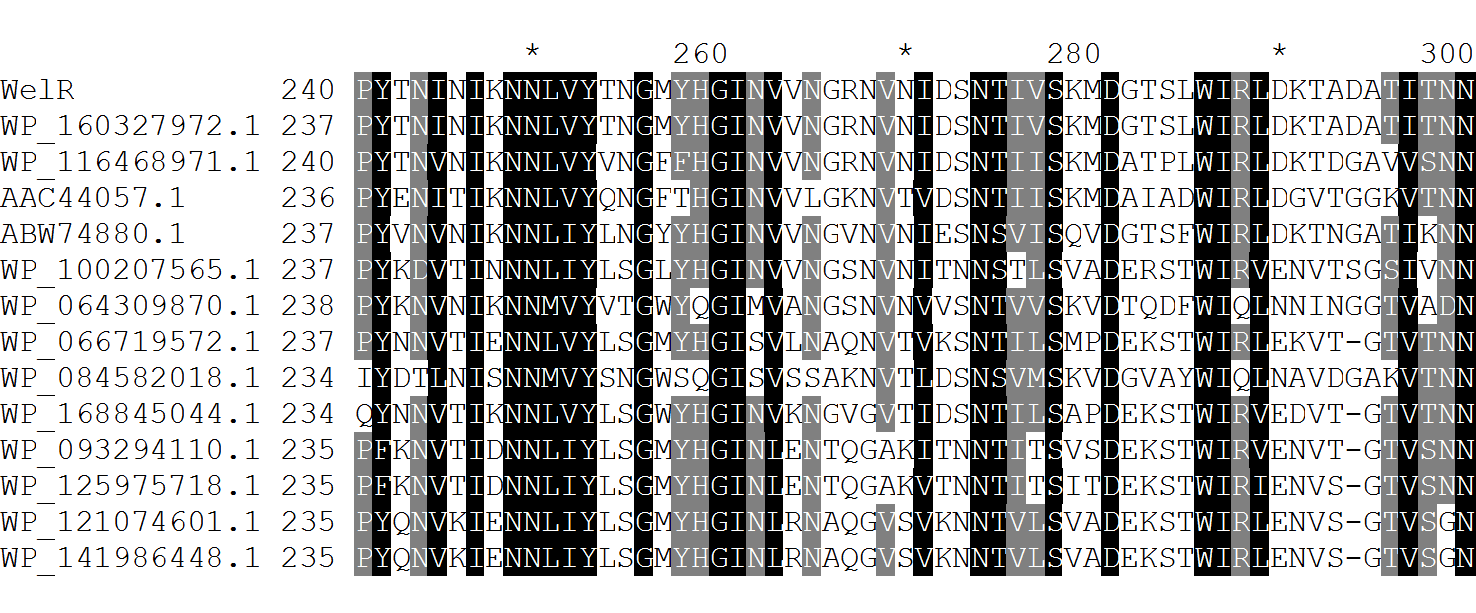

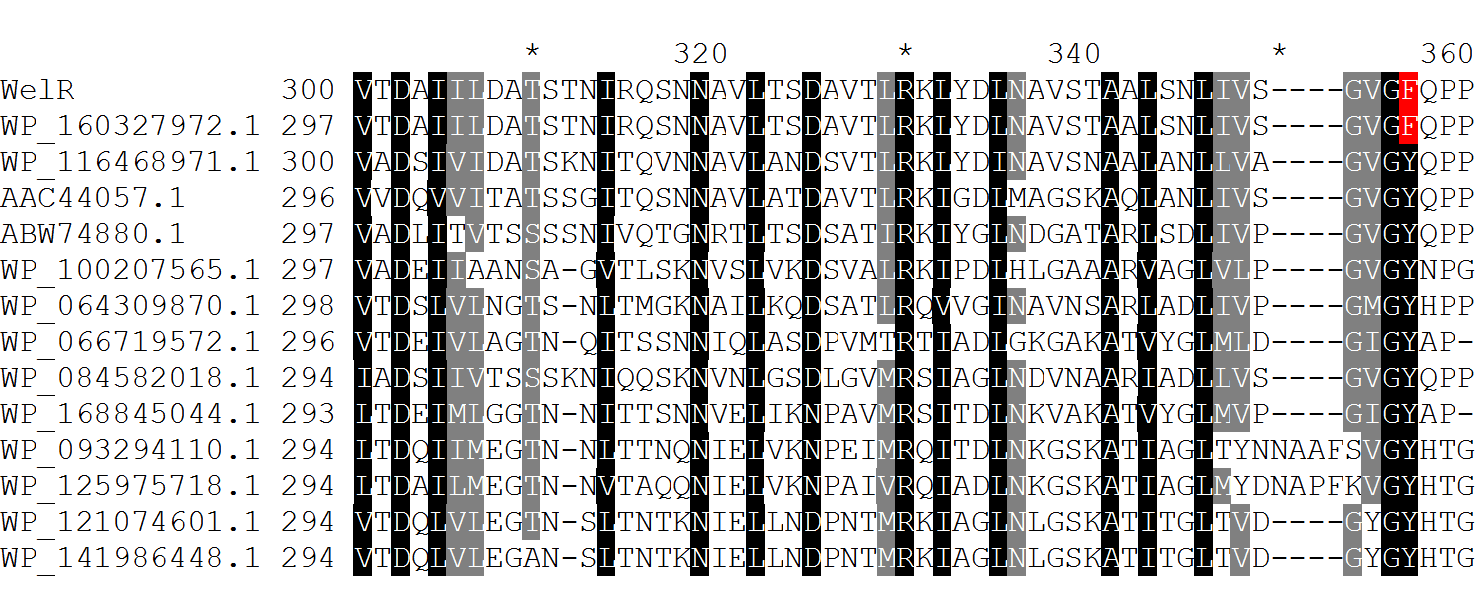

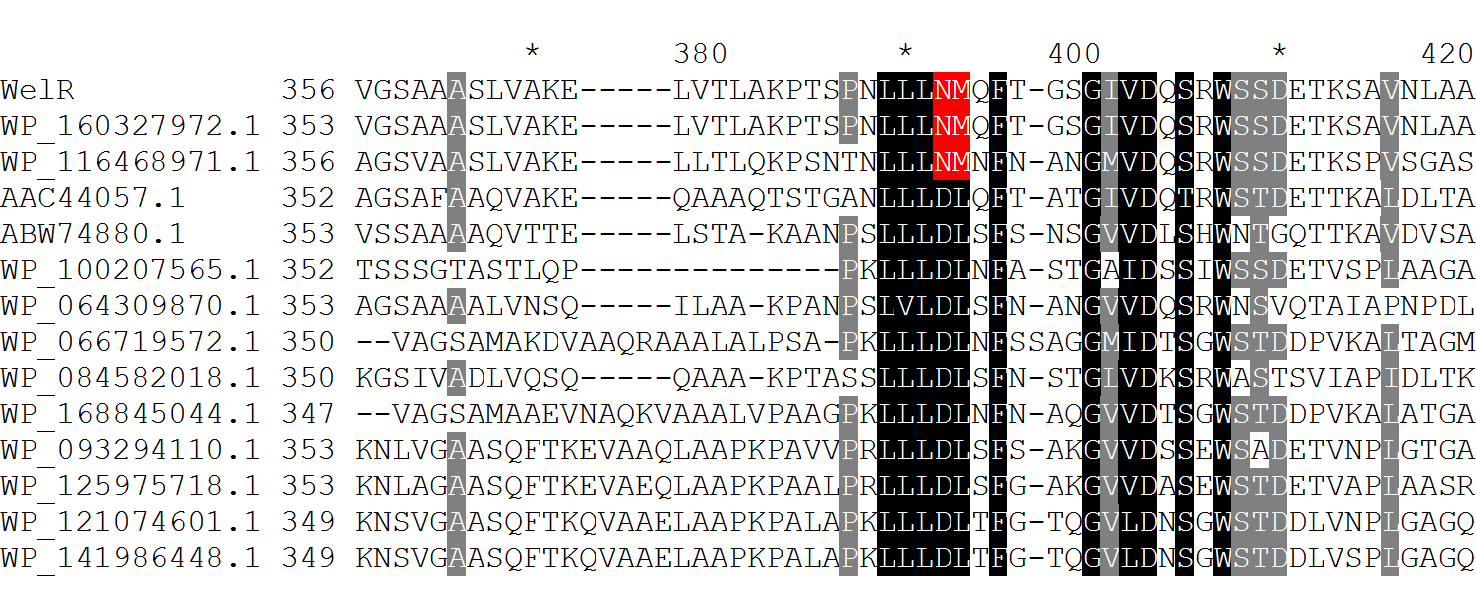

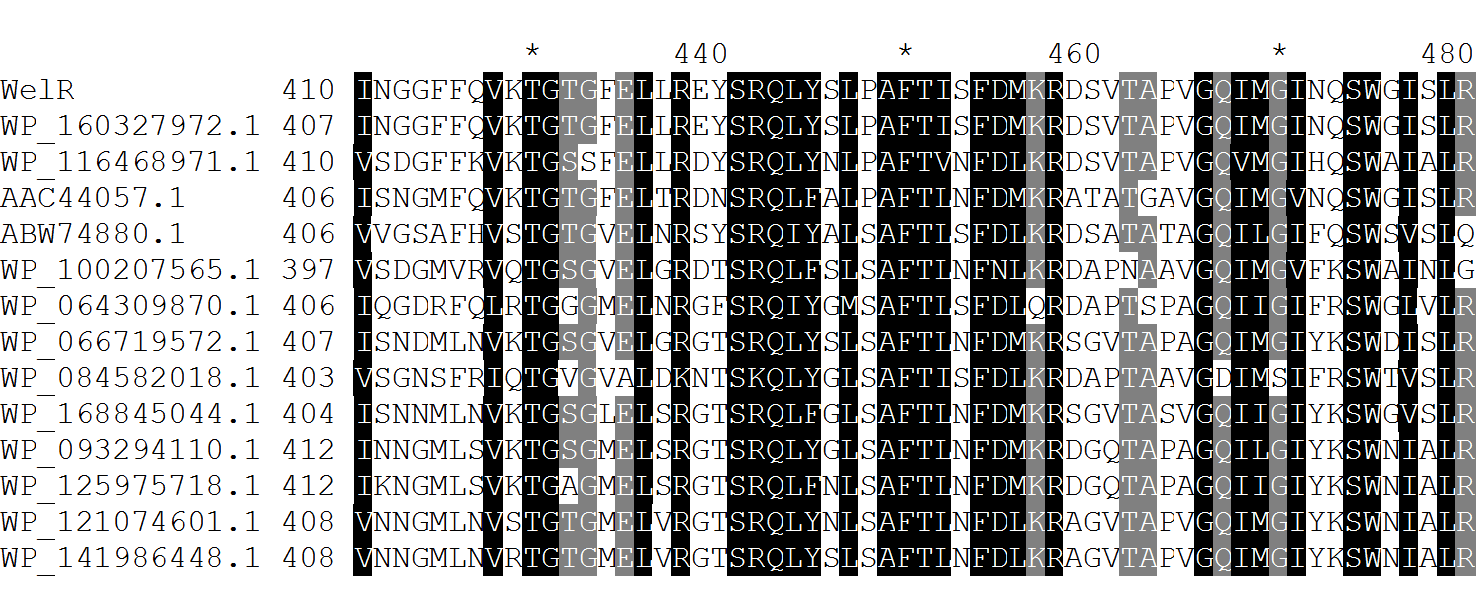

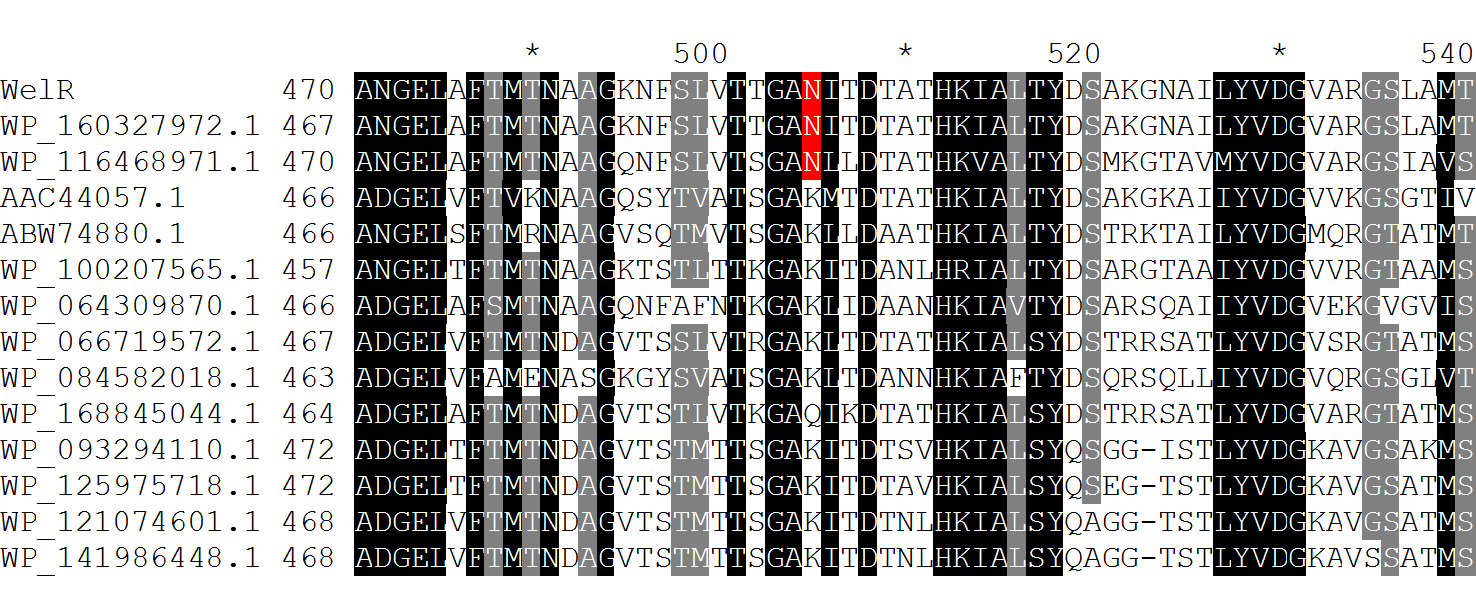

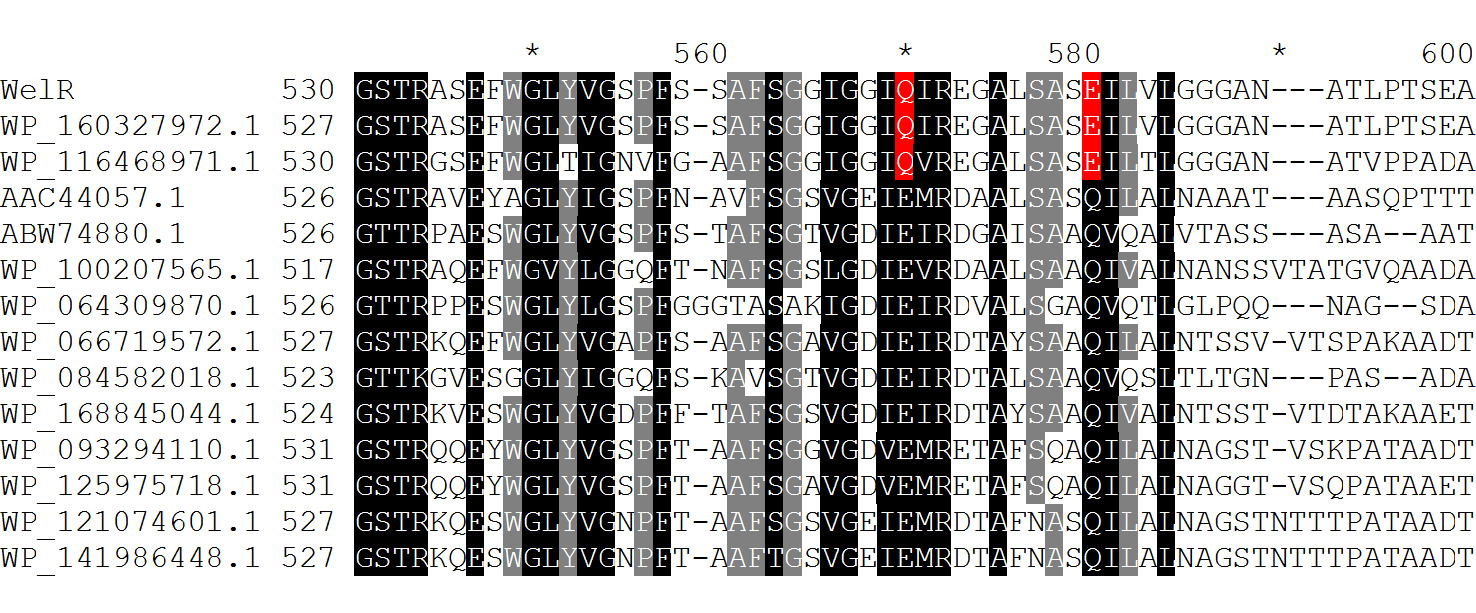

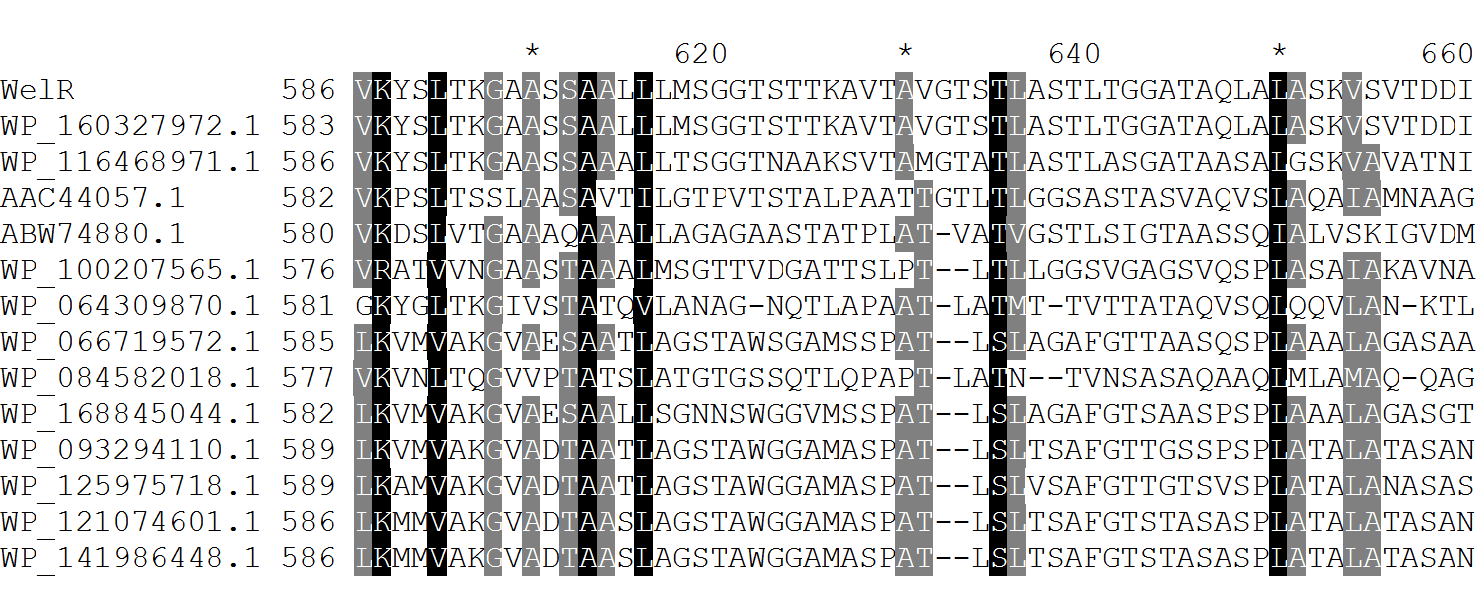

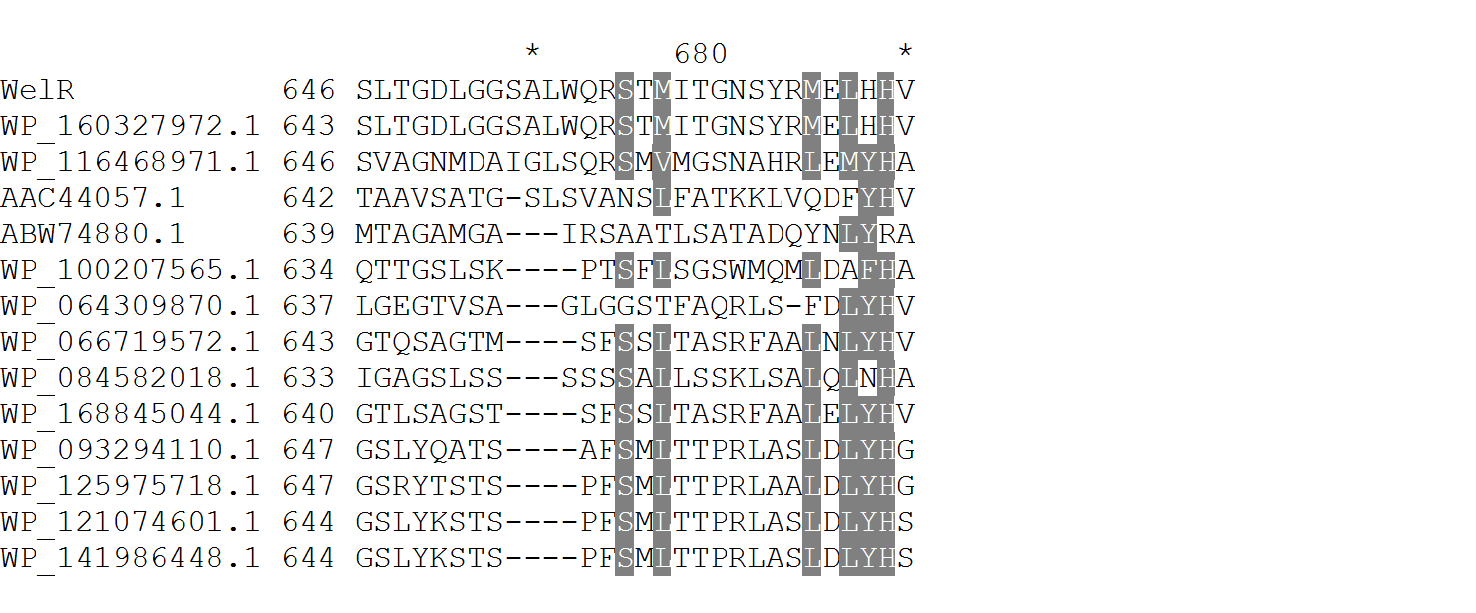
**

**Supplementary Figure 4.** Protein sequence alignments and differential amino acid sites of WelR. Protein sequences similar to WelR were retrieved from the GenBank database using the BLAST algorithm on the National Center for Biotechnology Information (NCBI) server (http://www.ncbi.nlm.nih.gov). The amino acid sequence was aligned with homologous sequences using the MEGA6 and ClustalX programs to predict key amino acids in the WelR and colored with GeneDoc program. 13 proteins sharing more than 40% homology with WelR were shown. 100% conserved amino acid residues are boxed in a dark shade, and amino acid residues above 80% conserve are boxed in a pale shade. The predicted key amino acids are shaded in red. 13 proteins are as follows: WP_160327972.1: right-handed parallel beta-helix repeat-containing protein of unclassified *Sphingomonas*; WP_116468971.1: hypothetical protein of *Sphingomonas pokkalii*; AAC44057.1: unknown protein of *Sphingomonas* sp. S88; ABW74880.1: putative diutan polysaccharide lyase of *Sphingomonas* sp. ATCC 53159; WP_100207565.1: hypothetical protein of *Sphingomonas* *elodea*; WP_064309870.1: hypothetical protein of *Sphingomonas* sp. TDK1; WP_066719572.1: hypothetical protein of *Sphingomonas pituitosa*; WP_084582018.1: hypothetical protein of *Sphingomonas azotifigens*; WP_168845044.1: right-handed parallel beta-helix repeat-containing protein of *Sphingomonas* sp. S2M10; WP_093294110.1: hypothetical protein of *Sphingomonas* sp. NFR04; WP_125975718.1: hypothetical protein of unclassified *Sphingomonas*; WP_121074601.1: hypothetical protein of *Sphingomonas elodea*; WP_141986448.1: hypothetical protein of *Sphingomonas trueperi*.

**
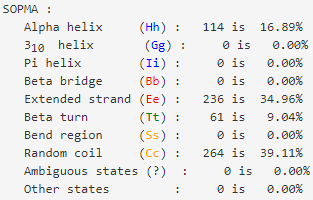

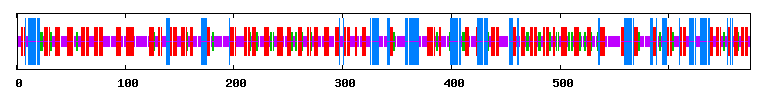
**

**Supplementary Figure 5.** Secondary structure prediction of WelR using the SOPMA server (htpts://npsa-prabi.ibcp.fr/cgi-bin/npsa_automat.pl?page=/NPSA/npsa_sopma.html) online.

**
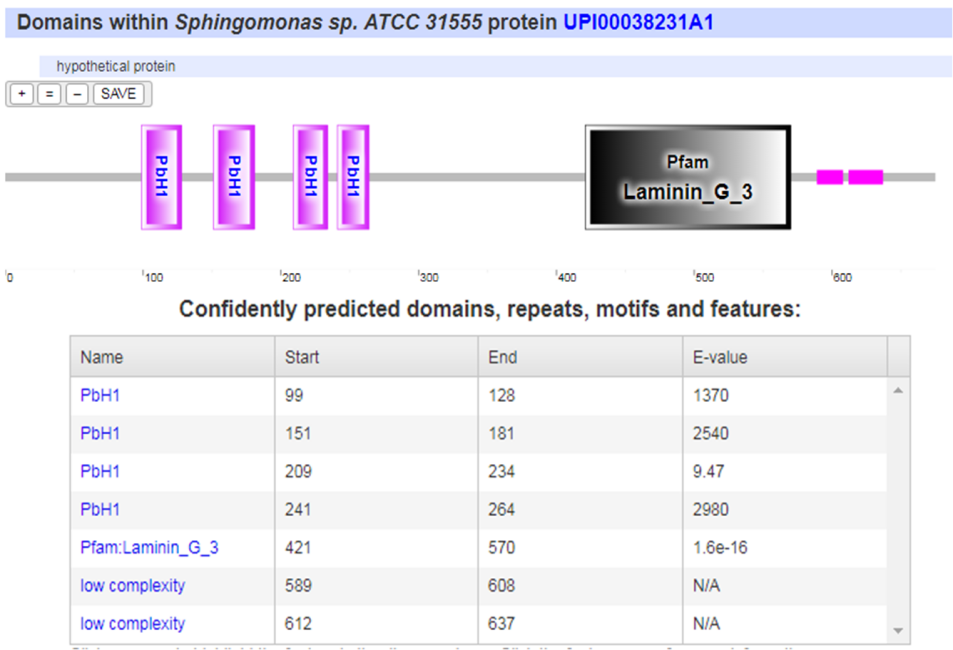
**

**Supplementary Figure 6.** Protein conserved domain prediction of WelR using the Simple Modular Architecture Research Tool (SMART) on the SMART server (http://smart.embl-heidelberg.de/) online.

**
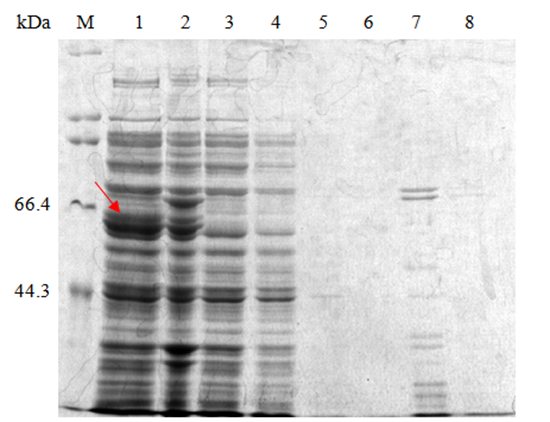
**

**Supplementary Figure 7.** Expression and purification of mutant protein Q558A. M: Molecular weight marker; Lane 1 to 2: soluble protein and insoluble protein of *E. coli* BL21/pET28a^(+)^-Q558A induced by 0.4 mM IPTG at 16 °C for 20 h; Lane 3: flow-through; Lane 4-6: 50 mM imidazole elution buffer; Lane 7: 100 mM imidazole elution buffer; Lane 8: 300 mM imidazole elution buffer.

## Supplementary Tables

**Supplementary Table 1.** All primers used in this study.

| **primer** | **5’-oligonucleotide sequence-3’** |
| --- | --- |
| *welR*-F1 | GGGAATTCATGCTTACCATGCCGGACG |
| *welR*-R2 | CCCAAGCTTTCAGACGTGGTGCAATTCC |
| N494AF-1 | TACCACCGGCGCGGCCATCACGGACACG |
| Q558AF-1 | ATCGGCGGCATTGCGATACGCGAAGG |
| E568AF-1 | CTGAGTGCTTCGGCGATCCTCGTCCTC |
| L19AR-2 | CTCGCTGCCTTCGCGGCAGCATCAAG |
| F352AR-2 | CCGACCGGCGGCTGGGCACCGACGCCGCTGAC |
| F352AF-1 | GTCAGCGGCGTCGGTGCCCAGCCGCCGGTCGG |
| N382AR-2 | CGGTAAACTGCATGGCTAGCAACAGGTTCGG |
| N382AF-1 | CCGAACCTGTTGCTAGCCATGCAGTTTACCG |
| M383AR-2 | CTGCCGGTAAACTGCGCGTTTAGCAACAGGTTC |
| M383AF-1 | GAACCTGTTGCTAAACGCGCAGTTTACCGGCAG |
| 16SqPCRFor | CAGCGTTTGACATGGTAGGAC |
| 16SqPCRRev | TAACCCAACATCTCACGACAC |
| welRqPCRFor | CACATCGAGCAGCAACATCG |
| welRqPCRRev | AGCATCCAGTCTTGTTCGGG |

**Supplementary Table 2.** The amino acid composition of WelR.

| **Amino acid** | **Composition** | **Hydrophobic parameter** |
| --- | --- | --- |
| Ala(A) 63 | 9.3% | 1.800 |
| Arg(R) 23 | 3.4% | -4.500 |
| Asn(N) 57 | 8.4% | -3.500 |
| Asp(D) 36 | 5.3% | -3.500 |
| Cys(C) 0 | 0.0% | 0 |
| Gln(Q) 21 | 3.1% | -3.500 |
| Glu(E) 16 | 2.4% | -3.500 |
| Gly(G) 63 | 9.3% | -0.400 |
| His(H) 9 | 1.3% | -3.200 |
| Ile(I) 44 | 6.5% | 4.500 |
| Leu(L) 64 | 9.5% | 3.800 |
| Lys(K) 25 | 3.7% | -3.900 |
| Met(M) 18 | 2.7% | 1.900 |
| Phe(F) 20 | 3.0% | 2.800 |
| Pro(P) 14 | 2.1% | -1.600 |
| Ser(S) 72 | 10.7% | -0.800 |
| Thr(T) 62 | 9.2% | -0.700 |
| Trp(W) 6 | 0.9% | -0.900 |
| Tyr(Y) 13 | 1.9% | -1.300 |
| Val(V) 49 | 7.3% | 4.200 |

**Supplementary Table 3.** The Cq values of welR and 16S rRNA.

| **1** | **16S rRNA Cq** | ***welR* Cq** |
| --- | --- | --- |
|  | 11.49 | 22.55 |
| Control group (12 h) | 11.44 | 22.48 |
|  | 11.69 | 22.46 |
|  | 13.85 | 22.20 |
| Test Group (48 h) | 13.89 | 22.29 |
|  | 14.09 | 22.46 |
| **2** | **16S rRNA Cq** | ***welR* Cq** |
|  | 9.90 | 22.07 |
| Control group (12 h) | 9.86 | 22.10 |
|  | 9.78 | 22.10 |
|  | 10.62 | 21.31 |
| Test Group (53 h) | 10.55 | 21.41 |
|  | 10.68 | 21.41 |
